# The Use of Enamel Matrix Derivative during Surgical Therapy for Peri-Implantitis: A Case Series

**DOI:** 10.3390/dj12010011

**Published:** 2023-12-30

**Authors:** Thomas G. Wilson, Stephen K. Harrel, Martha E. Nunn

**Affiliations:** 1 Private Practice of Periodontics, Dallas, TX 75231, USA; tom@northdallasdh.com; 2 College of Dentistry, Texas A&M University, 4510 Ridge Road, Dallas, TX 75229, USA; 3 Private Practice Dentistry and Biostatistics, Omaha, NE 68178, USA; menunn@gmail.com

**Keywords:** peri-implantitis, enamel matrix derivative, guided tissue regeneration, dental implant

## Abstract

Peri-implantitis is a growing concern and currently, there is no agreement on the best method for treating this condition. This study looked at surgical intervention with the use of enamel matrix derivative (EMD) for treating this condition. A cohort of 25 (34 implants) consecutive patients treated with EMD for peri-implantitis was followed for up to 6.4 years. The survival of the implants as well as changes in clinical parameters are reported. Statistical analysis was performed using paired t tests and general estimating equations. The mean length of time implants were followed post-surgery was 3.05 ± 1.53 years. All but two of the treated implants survived in function (94%). Both failed implants were lost in the same patient, who was a heavy smoker. The changes in mean probing depth (1.94 ± 1.18 mm), change in deepest probing depth (3.12 ± 1.45 mm), and reduction in bleeding on probing (73.6 ± 43.9%) according to patient means were all highly significant (*p* < 0.001 for all changes). When EMD is used during surgical treatment of peri-implantitis, there is a high survival rate of implants and significant improvements in clinical parameters.

## 1. Introduction

The use of enamel matrix derivative (EMD) (Emdogain(R), Institut Straumann AG, Basel, Switzerland) to treat periodontitis is well described and largely successful [1]. Significantly less information exists about the use of EMD around dental implants suffering from peri-implantitis. Peri-implantitis has been defined as a pathological condition occurring in tissues around dental implants, characterized by inflammation in the peri-implant mucosa and progressive loss of supporting bone [2]. Recommendations from a Workshop published in 2018 standardized this definition and it has been widely accepted [3]. Prior to standardization, the prevalence of peri-implantitis and appropriate treatment were difficult to establish due to varying definitions. Previous estimates of the extent of peri-implantitis have ranged from 6.61% [4] to 59% [5]. One recent study noted that the percentage of peri-implantitis was reduced when the 2018 criteria were used [6]. Regardless of the prevalence, due to the rapid increase in the number of implants in function, peri-implantitis is a growing problem for both patients and professionals.

The etiology of peri-implantitis is plaque-associated, characterized by inflammation of the peri-implant tissue, eventually leading to the loss of supporting bone [2].The etiology of bone loss was initially attributed to the same factors seen in periodontitis, an inflammatory lesion of the peri-implant soft tissues that precedes bone loss, with the primary etiologic factor being bacterial biofilm. This led to therapies that mirrored classic treatments for periodontitis. These ranged from non-surgical [7], to minimally invasive with minimal surface alteration [8], to the use of large flaps and multiple materials along with extensive altering of the implant surface [9]. While the results of these treatments have been mixed, surgery is reported to produce better long-term outcomes compared to closed procedures [10,11,12].

EMD has been found to exert a positive influence on soft tissue wound healing and the control of inflammation [13] and to have an antibacterial effect on in vivo dental biofilm [14]. One study showed that the use of EMD in combination with micro-spherical minocycline application was an effective adjunct for the treatment of peri-implant mucositis [15].

The effect of EMD on bone growth on titanium surfaces has been found to be dose-dependent. Schwarz and coworkers placed varying strengths of EMDs on sandblasted acid-etched titanium surfaces and compared the growth of human osteoblast-like cells to controls without EMD [16]. They concluded that EMD enhanced cell proliferation and viability of the osteoblasts compared to controls, and higher dosages resulted in more positive findings. Qu and coworkers placed osteoblast-like cells on course-grit-blasted and acid-attacked surface (SLA) titanium disks. They found that EMD increased the alkaline phosphatase activity and osteocalcin production and enhanced mRNA expression of osteoprotegerin (OPG) and did not influence that of RANKL [17].

In a human study, Mercado et al. [18] defined peri-implantitis as probing depths of 4 mm or greater with minimum bone loss of 20% after at least two years in function. Following elevation of full-thickness flaps and cleaning the implant surface with an ultrasonic scaler at low power, 24% EDTA (PrefGel(R), Institut Straumann AG, Basel, Switzerland) was applied to the implant surface for 2 min, and grafts using a combination of deproteinized bovine bone material, EMD, and doxycycline were placed. At 3 years post operative, all treated implants were still in place (100%), mean probing depth (PD) improved from 8.9 mm (±1.9) to 3.50 mm (±0.50) and bone loss from 6.92 mm (±0.50) to 2.60 mm (±0.73). A large percentage of the therapy was considered successful according to the Successful Treatment Outcome Criterion. Fifty-six percent had PDs less than 5 mm, no further bone loss, no bleeding on probing (BOP), and no recession after 36 months.

The results of a randomized clinical trial investigated 29 implants in humans diagnosed with peri-implantitis were reported in two papers. All were treated with an open-flap approach. The implant surfaces were debrided with gauze soaked in normal saline. The test group then had EMD placed, while the control group did not. At one year, multivariate modeling found that there was an increase of marginal bone associated with the use of EMD, the number of osseous walls, and a Gram-positive aerobic microbial flora. At 3 years, 100% of the test group implants survived, compared to 83% of the control group [19]. Members of this research group later reported the 5-year data on 25 of the original 29 implants in the study. At 5 years, 85% of the test group and 75% of the control group implants survived. They concluded that EMD was positively associated with implant survival compared to those that did not receive it [20].

The current case series reports on all implants surgically treated for peri-implantitis within a large implant database developed from the records of a single private practice of periodontics. All treatments used EMD as part of the surgical therapy. The primary aim was to determine the survival of the implants over time. Secondary aim were to report on the clinical conditions of the implants at the most recent postoperative visit.

## 2. Materials and Methods

This is a retrospective case series taken from data in a single private practice of periodontics. All procedures were performed by Board-Certified periodontists. All examiners were calibrated and standardized. The study was performed in accordance with the Declaration of Helsinki, Good Clinical Practices, and was registered with ClinicalTrials.gov, identifier NCT05419102. The protocol was approved by the Center for IRB Intelligence (Pro00059785) and complied with HIPAA requirements.

From an existing database of over 1800 implants placed or treated in the practice, data on all consecutive implants surgically treated for peri-implantitis between January 2015 and December 2020 were collected. Data collected included patient medical status, type of implant, type of restoration, date implant was treated, incision design (minimally invasive or larger flap), videoscope usage, percentage of bone loss (surgical estimate), re-evaluation date, use of hard tissue grafts, membranes, placement of connective tissue grafts, probing pocket depth before treatment and at re-evaluation (6 sites per implant), length of time since treatment, and bleeding on probing (4 sites per implant). EMD, following the manufacturers’ instructions, was used during the treatment of all the cases surgically treated for peri-implantitis.

A search of the database yielded a total of 34 implants in 25 patients that were treated for peri-implantitis with the use of EMD. The length of postoperative follow-up was from 0.71 to 6.4 years (mean ± SD: 3.05 ± 1.53 years). Fifteen of the implants were placed within the private practice treating the peri-implantitis, while nineteen were placed outside of the practice and referred for treatment.

This case series is reported in line with the PROCESS 2020 guidelines [21].

### Statical Analysis

Summary statistics were calculated for patient-level means of initial mean probing depth, final mean probing depth, initial deepest probing depth, final deepest probing depth, change in mean probing depth, change in deepest probing depth, initial mean bleeding on probing, final mean bleeding on probing, and change in mean bleeding on probing. Paired t tests for change in patient means of mean probing depth, deepest probing depth, and bleeding on probing were conducted. Frequencies were tabulated for all categorical measures without patient averaging. Similarly, summary measures were calculated for implant length and implant diameter without patient averaging.

Because of the lack of independence among implants within the same patient, the method of generalized estimating equations (GEE) was utilized to calculate adjusted means for mean probing depth and deepest probing depth while adjusting for the time between surgery and final follow-up for each variable. GEE binary modeling was also used to calculate odds ratios for the likelihood of improvement in bleeding on probing (BOP) for each variable while adjusting for time between surgery and final follow-up. An exchangeable working correlation was utilized for all GEE modeling.

## 3. Results

A cohort of 25 (34 implants, 11 male and 14 female) consecutive patients treated with EMD for peri-implantitis was followed for up to 6.4 years. Summary statistics calculated for patient-level means are shown in Table 1. Based on paired *t* tests of change in patient means of mean probing depth, deepest probing depth, and bleeding on probing, all measures changed significantly from initial measures to final measures. Frequencies of categorical measures are shown in Table 2.

GEE linear modeling was utilized to calculate adjusted means for mean probing depth and deepest probing depth while adjusting for the time between surgery and final follow-up for each variable. The results of these analyses are shown in Table 3.

GEE linear modeling for change in mean probing depth and deepest probing depth with only time between surgery and final measure was conducted, with time between surgery and final measure failing to be statistically significant for both models (*p* = 0.690 for mean probing depth model; *p* = 0.760 for deepest probing depth model), which indicates that changes in mean probing depth and deepest probing depth were stable over the course of the study. GEE binary modeling was also used to calculate odds ratios for the likelihood of improvement in bleeding on probing (BOP) for each variable while adjusting for time between surgery and final follow-up. Only one patient with two implants had diabetes mellitus, so estimation and testing of this variable is of limited inference. Similarly, only two patients with three implants had restorations that were unstable, so that estimation and testing is of limited inference. Based on GEE modeling, the only significant finding was for mean probing depth, with implants in the molar area demonstrating significantly less reduction in mean probing depth than implants in a non-molar location. Based on GEE estimation, there did appear to be potential trends according to whether an implant restoration was cemented or screwed, % bone loss, implantoplasty, and osseous reduction. A larger sample would be necessary to determine whether these potential trends are real or random. The fact that the change in mean probing depth, change in deepest probing depth, and reduction in bleeding on probing according to patient means were all highly significantly different (*p* < 0.001 for all changes) would seem to validate the lack of statistical findings from the GEE modeling.

Because only two implants in one patient, who was a smoker, failed, survival analysis was not possible. With a larger sample and more patients with failed implants, a survival analysis could be conducted. Having said this, the analysis of these data demonstrates a high rate of survival (94% survival) for implants treated for peri-implantitis where EMD with or without a bone graft was used.

## 4. Discussion

The results of this case series demonstrate a high level of survival (94%) of implants when EMD was applied during surgical treatment for peri-implantitis. Only two implants were lost in the cohort, and both were in a long-term heavy smoker. Further, there was a highly significant improvement in PD postoperatively and reduction in BOP when EMD was used. A significant improvement in PD was present over the entire postoperative period of 0.71 to 6.4 years (mean ± SD: 3.05 ± 1.53 years), with statistical analysis showing no difference in this trend over time.

Non-surgical treatment for peri-implantitis has not proved routinely successful [22]. The cohort of patients in this study was taken from a single private periodontal practice who had titanium dental implants that had been diagnosed with peri-implantitis and treated surgically. Part of each surgery consisted of minimal flap elevation and the placement of enamel matrix derivative (EMD). Because all implants were treated in a private practice over an extended period of time, there were variables in treatment protocols, post-treatment oral hygiene, and compliance with periodontal maintenance. Also, the lack of a control group for comparison is a limiting factor. However, when compared to other published studies, the implant survival improvement appears favorable when EMD is used. [23,24].

One of the goals of the surgery described here was to gain access for the removal of any foreign bodies in the tissue, including excess cement and particles of titanium. These foreign bodies have been suggested as possible etiologic factors in continued bone loss around implants [25,26,27,28]. It is of interest that 88% of the peri-implantitis affected implants in this study had cemented restorations, which may have been a source for foreign body particles. Visualization was aided in 20 of the cases (59%) by the use of a videoscope. In this practice, a videoscope is now used routinely in the treatment of peri-implantitis because it allows for minimal removal of the native implant surface, thus preserving the cellular attraction created by these surfaces [8]. The videoscope allows for the use of very small, minimally invasive incisions while facilitating precise removal of connective tissue, routinely containing cement or titanium particles, while minimizing surface damage.

**Figure 1:**
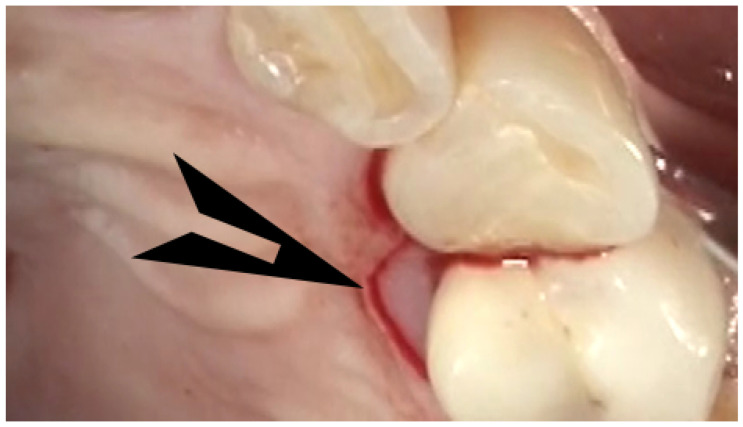
The minimally invasive surgical incisions for peri-implantitis currently used in the studied database. The total incision for an 8 mm peri-implant pocket on tooth #5 is shown on the palatal aspect (no buccal incision). The arrow shows the portion of the tissue next to the implant that frequently contains inflammation-causing foreign particles. This tissue will be removed.

**Figure 2:**
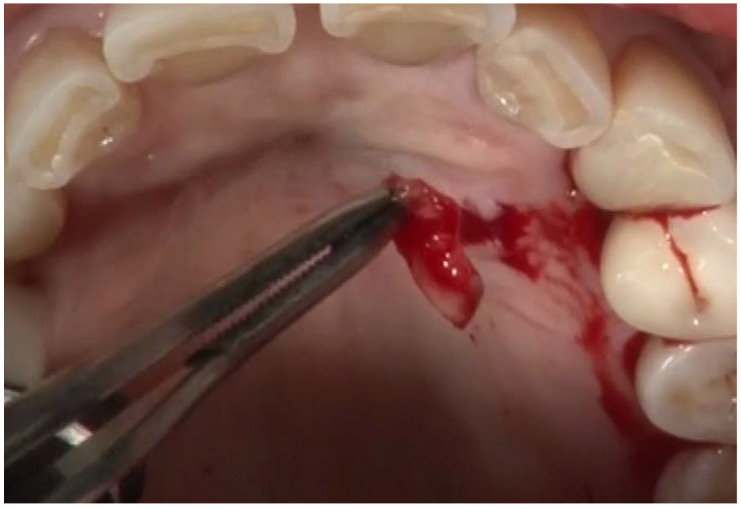
A minimally invasive flap on the palate has been elevated and the small piece of tissue potentially containing inflammatory foreign particles has been removed (held in forceps).

**Figure 3:**
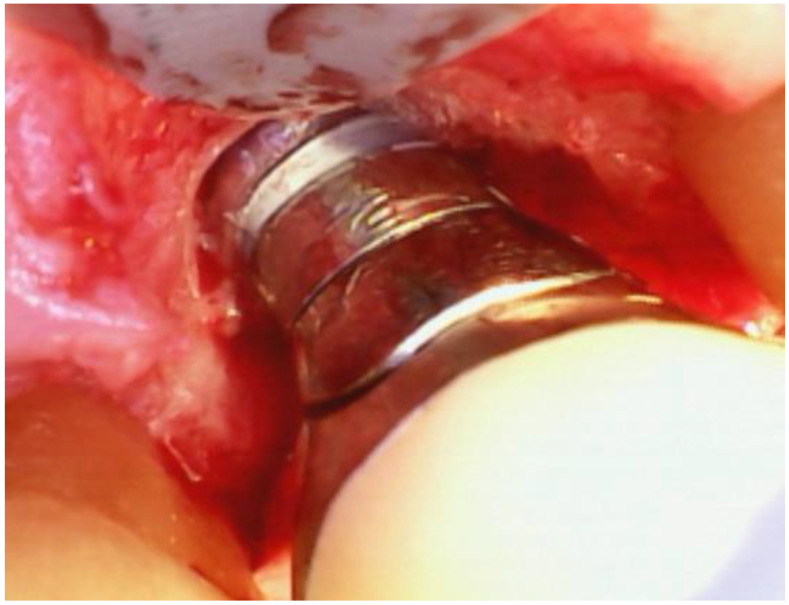
Videoscope image of the treated implant after removal of excess cement and granulation tissue. Note the exposed threads and osseous defect. The implant was subsequently disinfected with normal saline, and freeze-dried demineralized bone mixed with EMD was placed around the implant and in the osseous defect. Closure was achieved through isolated vertical mattress sutures on the mesial and distal of the implant.

As described in the previous paragraphs, the current methods used in the study practice are illustrated in Figure 1, Figure 2 and Figure 3. These figures illustrate the use of small, minimally invasive surgical access, the removal of the Ti- and cement-contaminated connective tissue adjacent to the implant, the use of the videoscope to visualize the surgical site using very small incisions, the use of saline on cotton to decontaminate the implant surface and maintain the remaining Ti oxide layer, the mixing of EMD with a freeze-dried bone allograft, and the placement of the graft EMD mixture in the bone defect. Because the treatment of peri-implantitis has evolved over time, EMD was used with all reported study implants, but not all of the steps illustrated in the figures were used with each study implant.

There are several shortcomings in this report. As in all studies that report on routine outcomes from a private practice, there were no control implants for comparison. Also, there was no data on soft tissue recession or the specific configuration of the osseous defects on the implants. There were many variables in the surgical and disinfection techniques used, with the only unifying factor being the use of EMD as part of the procedure. Therefore, it is not possible to say that the use of EMD alone was solely responsible for the high retention rate and favorable clinical outcomes reported.

## 5. Conclusions

These data demonstrate a high rate of survival and favorable improvement in clinical parameters for implants affected by peri-implantitis where EMD is used during surgery. The techniques for the treatment of peri-implantitis are rapidly evolving and there is a need for randomized controlled clinical trials to determine the most predictable approaches for therapy.

## Figures and Tables

**Table 1:** Summary statistics of patient mean periodontal measures.

	n	Mean	SD	Median	Min	Max	*p*
Mean Initial Probing Depth	25	4.56	1.01	4.6	3.1	8	<0.001
Mean Final Probing Depth	25	2.62	1.00	2.5	1.2	5.5	
Change in Mean Probing Depth	25	1.94	1.18	2.1	-0.5	4.4	
Deepest Initial Probing Depth	25	6.50	0.94	6	5	8	<0.001
Deepest Final Probing Depth	25	3.38	1.43	3	1.5	8	
Change in Deepest Probing Depth	25	3.12	1.45	3.5	−1	5	
Initial Bleeding on Probing	25	100.0%	0.0%	100.0%	100.0%	100.0%	<0.001
Final Bleeding on Probing	25	29.3%	45.5%	0.0%	0.0%	100.0%	
Reduction in Bleeding on Probing	25	73.6%	43.9%	100.0%	0.0%	100.0%	

**Table 2:** Demographics and implant characteristics.

Characteristic	%	Characteristic	%
**Immediate Implant?**		**Crown/Implant Stability?**	
Immediate	93.5% (29/31)	No	8.8% (3/34)
Not an immediate	6.5% (2/31)	Yes	91.2% (31/34)
**Cemented or Screwed Prosthesis?**		**Initial radiograph?**	
Cemented	88.2% (30/34)	No	0.0% (0/34)
Screwed	11.8% (4/34)	Yes	100.0% (34/34)
**Nightguard?**		**Final radiograph?**	
No Nightguard	67.6% (23/34)	No	20.6% (7/34)
Nightguard	32.4% (11/34)	Yes	79.4% (27/34)
**Diabetes Mellitus?**		**Implant Failure?**	
No Diabetes Mellitus	94.1% (32/34)	Survival	94.1% (32/34)
Diabetes Mellitus	5.9% (2/34)	Failure	5.9% (2/34)
**Smoker?**		**EMD + Bone?**	
Non-Smoker	91.2% (31/34)	EMD Only	57.6% (19/33)
Smoker	8.7% (3/34)	EMD + Bone	42.4% (14/33)
**Implantoplasty?**		**Implant Site Type**	
No	32.4% (11/34)	Upper Molar	26.5% (9/34)
Yes	67.6% 23/34)	Upper Premolar	11.8% (4/34)
**Osseous Reduction?**		Upper Cuspid	5.9% (2/34)
No	88.2% (30/34)	Upper Incisor	11.8% (4/34)
Yes	11.8% (4/34)	Lower Molar	26.5% (9/34)
**Minimally Invasive?**		Lower Premolar	11.8% (4/34)
No	41.2% (14/34)	Lower Cuspid	2.9% (1/34)
Yes	58.8% (20/34)	Lower Incisor	2.9% (1/34)
**Videoscope used?**		**Implant Length**	
No	58.8% (20/34)	Mean ± SD	9.97 ± 1.90
Yes	41.2% (14/34)	Median	10
**Soft tissue Graft?**		Range	6 to 13
No	76.5% (26/34)	**Implant Diameter**	
Yes	23.5% (8/34)	Mean ± SD	4.62 ± 1.43
**Bone Graft?**		Median	4.3
No	67.6% (23/34)	Range	3.3 to 11.5
Yes	32.4% (11/34)		
**Membrane?**			
No	67.6% (23/34)		
Yes	32.4% (11/34)		

**Table 3:** GEE means for mean probing depth and deepest probing depth adjusted for time from surgery to follow-up and odds ratio for BOP change adjusted for time from surgery to follow-up.

Variable	Change in Mean PD ± SE	*p*	Change in Deepest PD ± SE	*p*	OR for BOP Change (95% CI)	*p*
**Tooth Type**						
**Incisor**	3.03 ± 0.54	-	3.40 ± 0.48	-	NE	-
**Cuspid**	2.62 ± 0.45	0.279	4.24 ± 0.26	0.007		
**Premolar**	1.96 ± 0.24	0.071	2.76 ± 0.48	0.346		
**Molar**	1.58 ± 0.25	0.014	3.11 ± 0.38	0.640		
**Non-Molar or Molar**						
**Non-Molar**	2.41 ± 0.27	-	3.15 ± 0.35	-	1.00	
**Molar**	1.67 ± 0.26	0.022	3.17 ± 0.38	0.958	1.64 (0.24 to 11.4)	0.616
**Arch**						
**Mandibular**	1.98 ± 0.22	-	3.24 ± 0.35	-	1.00	
**Maxillary**	2.05 ± 0.32	0.845	3.10 ± 0.35	0.803	0.30 (0.05 to 1.85)	0.189
**Smoking Status**						
**Non-Smoker**	1.99 ± 0.22	-	3.18 ± 0.27	-	1.00	
**Smoker**	2.39 ± 0.90	0.672	2.92 ± 1.15	0.844	0.60 (0.02 to 19.0)	0.798
**Diabetes Mellitus**						
**No**	1.99 ± 0.23	-	3.15 ± 0.28	-	NE	-
**Yes**	2.58 ± 0.06 *	0.327	3.48 ± 0.07^*^	0.415		
**Cemented or Screwed**						
**Cemented**	1.97 ± 0.25	-	3.02 ± 0.30	-	NE	-
**Screwed**	2.36 ± 0.11	0.261	4.11 ± 0.39	0.128		
**Nightguard**						
**No**	1.98 ± 0.28	-	3.09 ± 0.38	-	NE	-
**Yes**	2.09 ± 0.35	0.813	3.32 ± 0.30	0.658		
**% Bone Loss**						
**<30%**	1.83 ± 0.36	-	3.27 ± 0.39	-	1.00	
**30% to <50%**	2.22 ± 0.23	0.293	3.49 ± 0.17	0.574	0.49 (0.05 to 4.65)	0.534
**50% and Greater**	1.73 ± 0.63	0.902	2.29 ± 0.84	0.284	0.11 (0.01 to 1.05)	0.056
**Implantoplasty**						
**No**	2.23 ± 0.27	-	3.67 ± 0.35	-	1.00	
**Yes**	1.90 ± 0.28	0.338	2.88 ± 0.33	0.137	0.77 (0.11 to 5.35)	0.788
**Osseous Reduction**						
**No**	1.99 ± 0.23	-	3.08 ± 0.30	-	1.00	
**Yes**	2.22 ± 0.38	0.579	3.73 ± 0.24	0.151	0.89 (0.08 to 10.4)	0.925
**Minimally Invasive**						
**No**	1.84 ± 0.31	-	3.35 ± 0.43	-	1.00	
**Yes**	2.17 ± 0.27	0.410	3.02 ± 0.33	0.534	0.52 (0.07 to 3.80)	0.491
**Videoscope**						
**No**	2.03 ± 0.32	-	3.08 ± 0.41	-	1.00	
**Yes**	2.00 ± 0.27	0.941	3.26 ± 0.35	0.746	2.17 (0.30 to 15.6)	0.423
**CT Graft**						
**No**	2.09 ± 0.30	-	3.00 ± 0.38	-	1.00	
**Yes**	1.82 ± 0.36	0.636	3.67 ± 0.34	0.278	2.11 (0.15 to 29.4)	0.540
**Bone Graft**						
**No**	2.10 ± 0.24	-	3.17 ± 0.30	-	1.00	
**Yes**	1.87 ± 0.40	0.605	3.15 ± 0.57	0.971	2.62 (0.25 to 27.5)	0.366
**Membrane**						
**No**	2.16 ± 0.28	-	3.27 ± 0.28	-	1.00	
**Yes**	1.69 ± 0.32	0.294	2.94 ± 0.60	0.654	0.93 (0.18 to 4.68)	0.929
**Stability (crown/implant)**						
**Stable**	2.00 ± 0.24	-	3.04 ± 0.28	-	NE	-
**Unstable**	2.16 ± 0.09 ^&^	0.583	4.46 ± 0.40 ^&^	0.153		
**EMD (with/without bone)**						
**EMD only**	1.98 ± 0.28	-	3.17 ± 0.35	-	1.00	
**EMD with bone**	2.03 ± 0.33	0.904	3.15 ± 0.45	0.973	4.75 (0.49 to 46.5)	0.119

* Two implants in one patient with diabetes mellitus. ^&^ Three implants in two patients that were unstable.

## Data Availability

Data is not available due to privacy concerns.
